# Die Another Day: Inhibition of Cell Death Pathways by Cytomegalovirus

**DOI:** 10.3390/v9090249

**Published:** 2017-09-02

**Authors:** Wolfram Brune, Christopher E. Andoniou

**Affiliations:** 1 Heinrich Pette Institute, Leibniz Institute for Experimental Virology, Hamburg 20251, Germany; 2 Immunology and Virology Program, Centre for Ophthalmology and Visual Science, the University of Western Australia, Crawley 6009, WA, Australia; 3 Centre for Experimental Immunology, Lions Eye Institute, Nedlands 6009, WA, Australia

**Keywords:** apoptosis, necrosis, necroptosis, HCMV, MCMV, vMIA, vICA, vIRA, vIBO

## Abstract

Multicellular organisms have evolved multiple genetically programmed cell death pathways that are essential for homeostasis. The finding that many viruses encode cell death inhibitors suggested that cellular suicide also functions as a first line of defence against invading pathogens. This theory was confirmed by studying viral mutants that lack certain cell death inhibitors. Cytomegaloviruses, a family of species-specific viruses, have proved particularly useful in this respect. Cytomegaloviruses are known to encode multiple death inhibitors that are required for efficient viral replication. Here, we outline the mechanisms used by the host cell to detect cytomegalovirus infection and discuss the methods employed by the cytomegalovirus family to prevent death of the host cell. In addition to enhancing our understanding of cytomegalovirus pathogenesis we detail how this research has provided significant insights into the cross-talk that exists between the various cell death pathways.

## 1. Introduction

The capacity to eliminate damaged or unwanted cells via regulated cell death programmes is essential for the wellbeing of multicellular organisms. Indeed, defective regulation of cell death pathways can result in the development of pathological conditions such as cancer, autoimmunity and inflammatory disease [1,2]. In addition to a role in embryonic development and tissue homeostasis [3], programmed cell death has the capacity to act as defence mechanism against invading pathogens [4,5]. The finding that many pathogens encode cell death inhibitors suggests that the ability to circumvent this process is essential for optimal replication and/or transmission of many pathogens.

Human cytomegalovirus (HCMV) is a large DNA virus that is common in the human population. After the initial acute infection, that is typically subclinical, HCMV establishes a latent infection that lasts for the lifetime of the host [6,7]. HCMV is known to employ an array of strategies designed to interfere with the host immune response and thereby prevent viral clearance. Since HCMV infection is strictly species-specific, animal models are often employed to study how virally-encoded genes contribute to viral pathogenesis in vivo. Murine CMV (MCMV), a natural mouse pathogen, shares a high degree of sequence homology and biology with HCMV making it an excellent model for HCMV infection. Targeted disruption of selected viral open reading frames has established that efficient viral replication depends on the capacity to inhibit the host cell death pathways. In this review we describe how CMV infection triggers a death response in the host cell, outline the countermeasures employed by CMV to prevent death, and detail how these contribute to in vivo viral pathogenesis.

## 2. Multiple Pathways to Death

Several genetically controlled cell death pathways are now recognised. Apoptosis is characterised by cell shrinkage, nuclear condensation, DNA fragmentation, and the formation of apoptotic bodies. The process is generally non-inflammatory since phagocytic cells rapidly engulf and digest the apoptotic bodies. Additional pathways to death such as necroptosis and pyroptosis have also been described. Unlike apoptosis, these necrotic forms of death are accompanied by cellular swelling, and ultimately rupture of the cell membrane, resulting in the release of cellular components into the extracellular space. Significant cross-talk between the different cell death pathways exist, allowing the distinct pathways to operate in a coordinated manner in order to promote immune responses to invading pathogens.

### 2.1. Apoptosis

The process of apoptosis may be initiated by either extrinsic or intrinsic signals. The intrinsic pathway is triggered in response to stimuli such as DNA damage, growth factor deprivation, or endoplasmic reticulum (ER) stress. Bcl-2 family proteins are the principle regulators of the intrinsic pathway that function by regulating the integrity of the mitochondrial outer membrane (MOM) [8]. MOM permeabilization (MOMP) results in the release of proteins such as cytochrome *c*, Smac/DIABLO, and Htr2/Omi that promotes the activation of caspases, a family of cysteine proteases responsible for mediating cellular destruction [9,10,11,12]. The Bcl-2 family is composed of three functional subgroups, BH3-only proteins that act as stress sensors and initiate apoptosis, the effector proteins Bax and Bak that mediate MOMP, and pro-survival proteins that maintain mitochondrial membrane integrity. Pro-survival Bcl-2 proteins have the capacity to bind to Bax and Bak and thus prevent their activation [8]. All pro-survival proteins appear capable of inhibiting Bax, while only Mcl-1, Bcl-x_L_ and A1 seem capable of holding Bak in check [13,14,15]. BH3-only proteins initiate apoptosis by binding to pro-survival Bcl-2 proteins and thereby releasing Bax and Bak [13,14]. Alternatively, some BH3-only proteins have the capacity to interact with Bax and Bak and directly catalyse their activation [16,17,18,19,20]. In healthy cells Bax and Bak exist as inert monomers, but as apoptosis proceeds the proteins undergo conformational changes resulting in the formation of large homo-oligomers that permeabilize the MOM. The Bcl-2 pathway therefore determines cell fate by regulating the activity of Bax and Bak. The importance of Bax and Bak to the apoptotic cascade was confirmed by the finding that cells isolated from Bax/Bak^−/−^ mice are highly resistant to many forms of apoptosis [21,22].

Death receptors (DR) may promote cell survival or death depending on the composition of the signalling complexes formed after receptor activation. DR are a subset of the tumour necrosis factor (TNF) receptor family characterised by a cytoplasmic domain of approximately 80 amino acids termed the death domain (DD). Fas-associated DD protein (FADD) is an adaptor protein that is critical for apoptotic signalling downstream of DR. Following activation of the DR Fas, DR4 or DR5, FADD is recruited to the receptor, via homotypic DD interactions, and FADD in turn, recruits initiator caspase-8 or caspase-10 [23,24]. Recruitment of the initiator caspases promotes the formation of dimers resulting in autocatalytic caspase activation [25]. Once activated, the initiator caspases promote apoptosis by cleaving effector caspases, such as caspase-3 and caspase-7, that then degrade critical cellular components. This process can be inhibited by the cellular FLICE inhibitor protein (cFLIP), a non-catalytic paralogue of caspase-8 (FLICE), that forms a heterodimer with caspase-8, thus preventing autocatalytic activation [26]. Initiator caspase activation, and hence apoptosis, may also result after TNF receptor 1 (TNFR1) activation. Ligand binding by TNFR1 results in the recruitment of the adaptors TNFR1-associated DD protein (TRADD) and TNFR-associated factor 2 (TRAF2), the receptor-interacting protein 1 (RIP1) kinase, and the cellular inhibitor of apoptosis protein (cIAP) into a signalling structure termed complex I. Formation of complex I promotes cell survival by activating the NF-κB pathway and inducing the production of pro-survival proteins [27]. Following internalization of complex I, complex II, a cytoplasmic signalling unit that includes the FADD adaptor and caspase-8 is formed. Several forms of complex II have been described with the stability and constituents of the complex regulated by an intricate series of ubiquitination event and phosphorylation events. Formation of complex II results in apoptosis only if autocatalytic processing of caspase-8 is able to take place, for example when cFLIP levels are low [28].

### 2.2. Necroptosis

Receptor interacting protein kinase-1 and -3 (RIPK1 and RIPK3) are key mediators of necroptosis with the study of signalling events downstream of TNFR1 being critical in understanding how these kinases promote necrotic death [29]. As outlined above, complex II is assembled in response to TNFR1 activation. Complex II, also called the “necrosome”, is composed of a heterodimer of caspase-8 and cFLIP along with FADD, RIPK1 and RIPK3. The presence of cFLIP in the complex prevents the autocatalytic activation of caspase-8, thus sparing the cell from apoptotic death. While caspase-8 is unable to undergo full catalytic activation when bound to cFLIP, a basal protease activity is present, and inhibition of caspase-8 activity has long been recognised as a method to promote necroptosis [30,31]. The finding that RIPK1 and RIPK3 are substrates of caspase-8 suggests that caspase-8 acts as a negative regulator of necroptosis by degrading and hence silencing the pro-necroptotic kinases [32,33]. Under circumstances where caspase-8 activity is silenced, RIPK1 recruits and activates RIPK3 through a RIP homotypic interaction motif (RHIM) [34,35,36]. Once activated, RIPK3 phosphorylates the mixed lineage kinase domain-like (MLKL) protein causing a conformational change within the MLKL that promotes its oligomerization. The oligomeric form of MLKL translocates to the plasma membrane where it interacts with the cell membrane and causes membrane rupture [37,38,39,40]. RIPK1-independent mechanisms for the activation of RIPK3 have also been described: the intracellular nucleotide sensor DNA-dependent activator of IFN-regulatory factors (DAI), also known as Z-DNA-binding protein 1(ZBP1), and the TIR-domain-containing adaptor inducing interferon-β (TRIF), both of which carry an RHIM, are capable of engaging RIPK3 and activating the necroptotic pathway [41,42].

### 2.3. Pyroptosis

The inflammatory caspases, that includes caspase-1, are critical for mediating innate defence. In response to cellular stress, inflammasomes are assembled that act as platforms for the activation of caspase-1. Activated caspase-1 promotes inflammation by cleaving pro-interleukin (IL)-1β and pro-IL-18 into their active forms, and initiates pyroptotic death by cleaving the gasdermin D protein. Once cleaved, the N-terminus of gasdermin D forms pores within the cell membrane causing the cell to swell and eventually results in membrane rupture [43,44,45]. The inflammasome is formed when pattern recognition receptors (PRRs), such as members of the Nod-like receptor family (NLR) or the absent in melanoma 2 (AIM2) protein, oligomerize in response to the detection of danger- or pathogen-associated molecules [46]. Clustering of the PRR results in the recruitment of the apoptosis-associated speck-like adaptor protein containing a CARD (ASC) that binds pro-caspase-1. The recruitment of pro-caspase-1 into the multi-protein assembly triggers proteolytic cleavage of pro-caspase-1 into its active form allowing the processing of downstream substrates. It should be noted that while caspase-1 is essential for the processing of pro-IL-1β and pro-IL-18, activation of caspase-11 can induce pyoptosis in some settings [47]. The inflammatory caspases therefore link the processes of inflammation and cell death induced following infection.

### 2.4. Cross-Talk between Death Pathways

The preceding description of the cell death pathways may give the impression that cell death results from the activation of linear signalling cascades. In fact, significant cross-talk between the various pathways exist that determines if a cell will die, and if so, by what mechanism. The interconnection of the pathways is exemplified by caspase-8 whose activation initiates apoptosis, while basal activity of uncleaved casapse-8 is essential to prevent necroptosis. Similarly, instances where activation of RIPK1 causes apoptosis, or promotes cell survival, rather than activating necroptosis have been reported. Antagonism of the ubiquitin ligases cIAP1 and cIAP2 is sufficient to result in the formation of the “Ripoptosome” a complex composed of FADD, caspase-8 and RIPK1 that assembles independently of DR signalling [48,49]. The kinase activity of RIPK1 is required for formation of the Ripoptosome with the complex mediating caspase-8 dependent apoptosis. The finding that RIPK1-deficent mice die shortly after birth suggested that in addition to its role in promoting death RIPK1 has a pro-survival function [50]. Inactivation of both caspase-8 and RIPK3 (or FADD and RIPK1) was required to prevent the lethality associated with RIPK1, indicating that FADD and caspase-8 promote survival by suppressing RIPK1 and RIPK3-mediated necroptosis during development [51,52,53]. Moreover, RIPK1 also has a kinase-independent pro-survival function under certain conditions [54,55,56].

The processes of apoptosis and pyroptosis are also linked. Procasapse-8 can be recruited to the inflammasome by interacting with the ASC adaptor protein leading to apoptotic death [57]. These observations demonstrate some of the ways in which the cell death pathways are linked. A feature of the interlocking regulation of these pathways is that inhibiting one cell death mechanism often results in the activation of an alternative pathway. An extension of this observation then is that ability of pathogens to inhibit the innate death response will require multiple inhibitory proteins.

## 3. Activation of Cell Death Pathways by CMV

In order for cell death to function as a defence mechanism, the host cell must have an effective means for detecting the presence of an invading pathogen. Cell death can be triggered when the host cell detects pathogen-associated molecular patterns (PAMPs), or can occur in response to cellular stress that is induced by the replication of intracellular pathogens. The following section describes some of the known mechanisms used by the host to detect CMV and how these can activate a cell death response.

### 3.1. Direct Recognition of CMV Infection

PRRs are a class of receptors that recognise molecules expressed, or produced by pathogens. Toll-like receptors (TLRs) were the first members of the PRR family to be identified with several implicated in the detection of CMV. A heterodimer of TLR1 and TRL2 was found to bind glycoproteins B and H produced by HCMV [58]. Whether MCMV can be detected by this mechanism has not been determined, but TLR2 knockout mice were found to be more susceptible to MCMV infection suggesting this pathway could be relevant during in vivo infection [59]. Increased titres of MCMV were also detected in mice lacking TLR3, which recognises double-stranded RNA (dsRNA), or TLR9, a receptor for unmethylated DNA [60,61]. TLR signalling typically results in the production of pro-inflammatory cytokines and chemokines. More recently, though, activation of many TLRs was found to result in the formation of signalling complexes capable of inducing RIPK-dependent necroptosis or caspase-8-dependent apoptosis [42,49,62].

In addition to TLRs, the DAI/ZBP1 and AIM2 sensors detect MCMV infection. Both of these PRR recognise cytoplasmic dsDNA, and both are activated during MCMV infection. Upon activation, DAI/ZBP1 can interact with RIPK3, promoting its activation that ultimately results in necroptosis [41]. More recent studies have shown that DAI/ZBP1 activation during MCMV infection requires viral gene transcription, but not viral DNA replication, and that DAI/ZBP1 is activated by the dsRNA in the Z-conformation (Z-RNA) rather than by the Z-DNA [63,64]. By contrast, the DNA sensor AIM2 suppresses MCMV replication via activation of the inflammasome [65]. While AIM2 was found to be required for the production of anti-viral cytokines in this context, the potential contribution of pyroptosis to the control of MCMV was not addressed. Activation of dsRNA-dependent protein kinase (PKR) is an additional mechanism by which CMV infection is detected [66,67]. PKR forms a homodimer and undergoes autophosphorylation after binding an RNA target resulting in catalytic activation of PKR. Once activated, PKR inhibits viral replication via several mechanisms, the best characterised of which is the inhibition of cellular protein translation by phosphorylation of the eukaryotic translation initiation factor 2α (eIF2α) [68]. How PKR activation mediates cell death has not been completely elucidated, but activation of NF-κB and/or FADD-dependent caspase-8 activation have been implicated [69,70]. PKR induces cell death in response to infection by influenza or poxviruses, but whether this process occurs following CMV infection has not been determined.

### 3.2. Cellular Stress Responses

Viral replication places a significant burden on the host cell, which responds by activating stress response pathways. CMV replication requires large amounts of protein to be synthesised. As a consequence, unfolded proteins accumulate within the ER resulting in the activation of a stress response after CMV infection [71,72]. The unfolded protein response is designed to restore ER homeostasis, however, if ER stress is not resolved cell death can result [73]. In response to ER stress the PKR-like ER kinase (PERK) phosphorylates eIF2α, resulting in the attenuation of mRNA translation, and in doing so, preventing the influx of additional protein into the stressed ER. Activation of PERK also allows for the preferential translation of proteins that contribute to the resolution of ER stress, or the induction of apoptosis, including the transcription factor C/EBP homologous protein (CHOP). A second mediator of ER stress responses is inositol-requiring enzyme 1 (IRE1), which undergoes oligomerisation in response to the accumulation of unfolded proteins within the ER. Once activated, IRE1 stimulates expression of ER-associated degradation factors and chaperones in order to alleviate ER stress. However, IRE1 can also promote apoptosis by activating a TRAF2-ASK1-JNK signalling cascade [73], by promoting caspase-12 or caspase-2 activation [74,75], or by interacting with Bax and Bak [76].

A second form of stress response triggered by CMV infection is the DNA damage response. Following detection of DNA damage, pathways that inhibit cell cycle progression are activated allowing for repair of the damaged DNA, or if the damage is too severe, apoptosis ensues [77]. Replication of the HCMV genome is achieved by a rolling-circle mechanism, and the resulting concatameric genomes are then cleaved to unit-length genomes [7]. This mechanism produces multiple exposed ends that can be recognised as dsDNA breaks. The ataxia-telangiectasia mutated (ATM) kinase pathway that responds to dsDNA breaks is activated following HCMV infection [78]. ATM has the capacity to activate p53 that can then induce pro-apoptotic proteins including Bax, Fas and the p53-induced protein with a DD (PIDD). HCMV replication also triggers a second type of DNA damage response activated in response to stalled replication forks [79]. The ATM-Rad3-related kinase (ATR) is the main transducer of this process, with p53 again acting as one of the downstream effectors for this pathway. Hence, replication of the viral genome, a process that is essential for viral propagation, activates several host cell defence mechanisms.

In summary, the host cell has the capacity to activate multiple cell death pathways either by directly detecting CMV constituents or indirectly by stress pathways activated by viral replication.

## 4. CMV-Encoded Death Inhibitors

Given the array of mechanisms that can lead to cell death in response to viral infection, a slow growing virus such as CMV would be expected to encode multiple death inhibitors. The ability to rapidly generate viral mutants lacking specific viral open reading frames has resulted in the identification of numerous CMV-encoded death inhibitors and aided in understanding how these proteins contribute to viral replication.

### 4.1. Inhibition of MOMP

**Figure 1:**
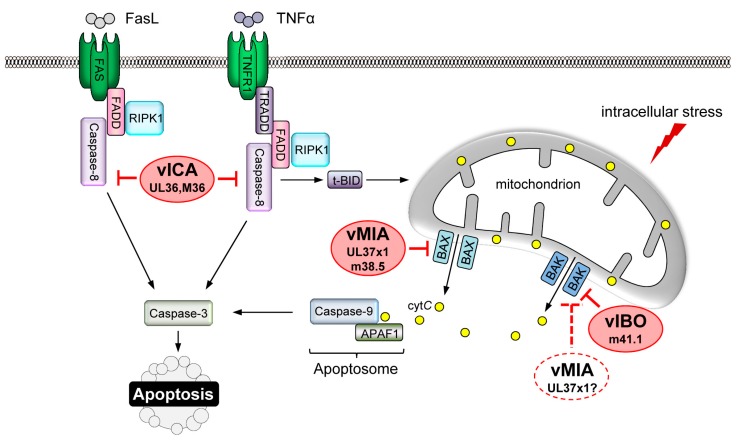
Inhibition of apoptosis by cytomegalovirus (CMV). The viral mitochondria-localized inhibitor of apoptosis (vMIA) and the viral inhibitor of BAK oligomerization (vIBO) inhibit mitochondrial outer membrane permabilization and release of proapoptotic factors (such as cytochrome *C* (cyt*C*)) by interacting with BAX and BAK, respectively. While murine CMV (MCMV) encodes two specific inhibitors, m38.5 and m41.1, human CMV (HCMV) has only one inhibitor, UL37x1. Whether the UL37x1 protein is BAX-specific or inhibits both BAX and BAK remains controversial. The extrinsic apoptosis pathway initiated by death receptor stimulation is blocked by the viral inhibitor of caspase-8 activation (vICA), which is encoded by the HCMV *UL36* and the MCMV *M36* gene, respectively. FasL: Fas ligand; FADD: Fas-associated death domain protein; RIPK1: receptor interacting protein kinase-1; TNFα: tumour necrosis factor α; TNFR1: TNF receptor 1; TRADD: TNFR1-associated death domain protein; t-BID: truncated BH3-interacting domain death agonist; APAF1: apoptotic protease activating factor 1.

A key step in the execution of intrinsic apoptosis is the permeabilization of the MOM by Bax and/or Bak. HCMV prevents MOMP by targeting Bax, and possibly Bak. The protein product of UL37 exon 1 (UL37x1) of HCMV, termed viral mitochondria-localized inhibitor of apoptosis (vMIA), binds and sequesters Bax at the mitochondrial membrane [80,81,82] (Figure 1). These early reports defined vMIA as a Bax-specific inhibitor, and suggested that activation of Bak requires the action of Bax in some cell types [81]. Thus, the inhibition of Bax by vMIA may be sufficient to prevent MOMP during HCMV infection. Several later studies have demonstrated an association between vMIA and Bak, implying that vMIA is capable of inhibiting both pro-apoptotic effector proteins [83,84]. If vMIA inhibits Bak, in addition to Bax, during viral infection remains to be confirmed. Several additional functions have been ascribed to vMIA. Expression of vMIA is sufficient to induce the release of calcium stores from the ER, a process that may modulate the apoptotic response [85]. In addition to its anti-apoptotic role vMIA inhibits anti-viral signalling downstream of the mitochondrial antiviral-signaling protein (MAVS) at mitochondria and peroxisomes [86,87]. vMIA therefore has the potential to promote viral replication via several mechanisms.

Unlike HCMV, MCMV encodes distinct inhibitors of Bax and Bak. The m38.5 protein of MCMV localises to mitochondria where it binds Bax and prevents its activation [84,88,89,90]. Although the MCMV m38.5 and HCMV UL37x1 proteins share little sequence similarity, they are very similar in their functions and their genes are located at analogous positions within the viral genomes. Therefore, m38.5 is also referred to as the vMIA of MCMV (Figure 1). A second MCMV-derived inhibitor, m41.1, associates with Bak at the mitochondrial membrane and acts as a viral inhibitor of Bak oligomerisation (vIBO) [91] (Figure 1). Cells infected in vitro with MCMV mutants lacking either m38.5 or m41.1 are sensitive to apoptosis induced by a range of stimuli [88,89,91,92]. Since activation of either Bax or Bak is sufficient to induce apoptosis, it is surprising that the in vivo growth characteristics of an Δm38.5 mutant differed from that observed when m41.1 was absent. Replication of a Δm41.1 mutant was attenuated in the liver and lungs, while deletion of m38.5 had no impact on viral replication at these sites [89,92,93,94]. By contrast, MCMV replication in leukocytes was reduced to a similar extent when either m38.5 or m41.1 was absent [89,92,93]. Optimal replication of MCMV therefore depends upon m38.5 and m41.1, whose combined activities maintain mitochondrial integrity. Overall the data suggest that inhibition of the intrinsic apoptotic pathway is an important requirement for CMV replication.

The perturbation of mitochondrial metabolism that occurs during viral infection can induce apoptosis. HCMV prevents cell death induced by oxidative stress by producing large amounts of a 2.7-kilobase non-coding RNA. During infection the β2.7 RNA interacts with complex I of the respiratory transport chain, resulting in maintenance of mitochondrial membrane potential [95]. Genes associated with retinoid/interferon-induced mortality (GRIM)-19 is an essential component of complex I that relocalises to a perinuclear region in response to oxidative stress [96]. β2.7 interacts with GRIM-19 and prevents its relocalisation from the mitochondria, allowing oxidative phosphorylation to continue and preventing oxidative stress-induced death [95].

### 4.2. Suppression of the ER Stress Response

The survival of CMV-infected cells depends on the ability to modulate the ER stress response. HCMV counteracts this process, in part, via the production of UL38. Cells infected with a HCMV mutant lacking UL38 die prematurely with cells displaying morphological changes consistent with the induction of apoptosis [97]. UL38 is a multifunctional protein with expression of the N-terminal 239 amino acids sufficient to suppress apoptosis [98,99]. Expression of UL38 is associated with accumulation of the activating transcription factor 4 (ATF4) and suppression of JNK activity [98]. The ATF4 transcription factor helps to resolve ER stress by inducing the production of proteins that facilitate protein folding within the ER. The inhibition of JNK activation prevents phosphorylation of Bcl-2 and Bim and so maintains the integrity of the mitochondrial membrane. Importantly, overexpression of ATF4 or inhibition of JNK activity reduced the death of cells infected with a pUL38-deficient virus demonstrating the functional relevance of these changes to the suppression of apoptosis [98]. The *M38* gene of MCMV shares significant homology with *UL38*, suggesting that MCMV might have conserved this mechanism for inhibiting ER stress-induced death, however, this has not yet been formally tested.

A second mechanism for manipulating the ER stress response by CMV is downregulation of IRE1 protein levels. The M50 protein of MCMV interacts with IRE1, causing its degradation at late times post infection [100]. UL50, the HCMV homologue of M50, was found to have a similar impact on IRE1 expression [100]. By inducing the degradation of IRE1 the M50/UL50 proteins should restrict all IRE1 signalling events, including the activation of apoptosis-inducing pathways. However, the impact of M50/UL50 on ER stress-induced apoptosis has not yet been investigated.

### 4.3. Inhibition of DR-Mediated Apoptosis

Multiple proteins involved in immune recognition including DR are downregulated following infection with CMV. Cell surface expression of the Fas and TNF receptors are reduced following infection with HCMV or MCMV, respectively [101,102]. Surprisingly, the UL138 protein encoded by HCMV increases TNFR1 levels at the cell surface [103,104]. While these changes have been noted following in vitro infection how they impact on viral replication in vivo is unclear. The impact of CMV infection on the expression of the TNF-related apoptosis-inducing ligand (TRAIL) receptors has been more extensively characterised. The MCMV m166 open reading frame inhibits the cell surface expression of the TRAIL receptor [105]. Importantly, the in vivo replication of an m166 deletion virus was compromised, an effect that could be overcome by depleting NK cells. Direct targeting of the TRAIL receptor therefore allows MCMV-infected cells to avoid elimination by innate immune effector cells. A similar pathway is likely to exist in humans since the UL141 glycoprotein of HCMV is capable of binding to TRAIL receptors and promoting receptor retention within the cell [106].

CMV-encoded proteins that interfere with DR signalling also contribute to viral pathogenesis. HCMV encodes the viral inhibitor of caspase-8 activation (vICA), the protein product of the *UL36* gene. HCMV vICA inhibits Fas-induced cell death by binding to pro-caspase-8 and preventing its activation [107] (Figure 1). Homologues of vICA have been identified in genomes of CMVs from different species implying that the capacity to inhibit DR signalling is an important requirement for CMV pathogenesis [108]. M36 is the vICA gene of MCMV. It is dispensable for viral replication in fibroblasts, but required for optimal replication in macrophages in vitro [109]. The replication defect of an MCMV M36 deletion mutant in macrophages was fully rescued by expression of HCMV UL36 or overexpression of a dominant-negative FADD [110,111] and partially rescued by expression of MC159, a viral FLIP of the molluscum contagiosum virus [112]. Remarkably, the in vivo growth defect of MCMV mutants lacking M36 was rescued by depletion of macrophages [113]. This finding is consistent with in vitro experiments where TNFα produced by macrophages induced apoptosis in cells infected with a ΔM36 virus. Thus, M36 allows MCMV-infected cells to resist the action of DR ligands produced by the host in response to infection. Similar to what has been observed with MCMV M36, HCMV UL36 was also required for cell death inhibition and viral replication in monocyte-derived macrophages [114]. However, cell death induced by a UL36-deficient virus could be inhibited by the broad-spectrum caspase inhibitor zVAD-fmk only at early but not at late times of monocyte-to-macrophage differentiation. This finding was interpreted as an indication that UL36 inhibits caspase-dependent as well as caspase-independent cell death programs in monocyte-derived macrophages [114].

### 4.4. Replication of CMV Requires Suppression of Necroptosis

**Figure 2:**
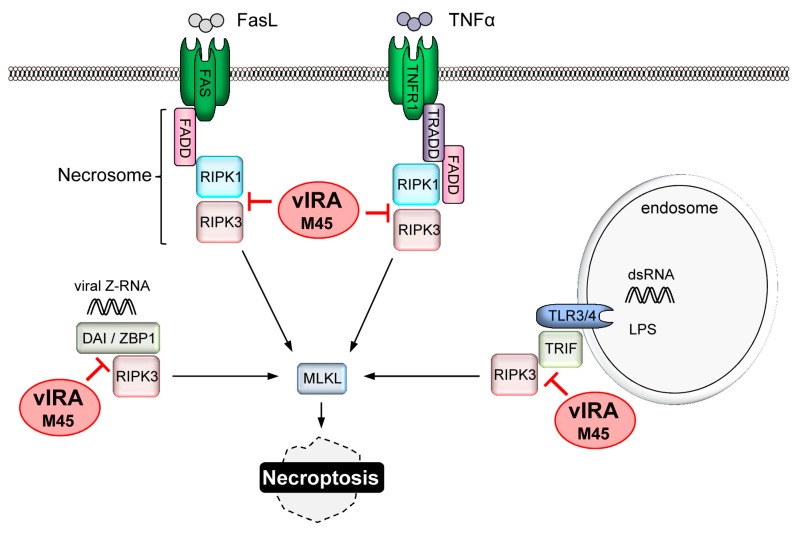
Inhibition of necroptosis by MCMV. Induction of programmed necrosis (necroptosis) involves activation of RIPK3 and mixed lineage kinase domain-like (MLKL). The viral inhibitor of RIP activation (vIRA), encoded by the MCMV *M45* gene, contains a RIP homotypic interaction motif (RHIM) and inhibits RHIM-dependent activation of RIPK3 by RIPK1, DAI/ZBP1, or TRIF. Death receptor-induced necroptosis requires caspase-8 inhibition, e.g., by vICA. LPS: lipopolysaccharide.

As mentioned previously, the necroptotic death pathway can be activated under circumstances where caspase-8 activity is suppressed. The action of vICA, while offering protection from DR-mediated apoptosis, could conceivably sensitise cells to necroptosis. Detection of MCMV infection by host PRRs constitutes an additional mechanism by which the necroptotic pathway could be activated. Analysis of MCMV mutants has established that the capacity to inhibit necroptosis is essential for viral replication in vivo. Screening of a random transposon library identified the M45 protein as being important for preventing the death of infected endothelial cells and macrophages in vitro, and that the activity of M45 is essential for in vivo replication [115,116]. M45 interacts with both RIPK1 and RIPK3 and prevents necroptosis initiated by Fas or TNFR activation in vitro [117,118]. Therefore, M45 has been termed viral inhibitor of RIP activation (vIRA) (Figure 2). Construction of an MCMV mutant bearing an inactivating mutation within M45 clarified the physiological relevance of these findings [119]. Knockdown of RIPK1 expression or pharmacological inhibition of RIPK1 activity was unable to prevent necroptosis induced by the M45 mutant virus in vitro establishing that MCMV infection activates necroptosis via a RIPK1-independent mechanism [119]. By contrast, growth of the M45 mutant virus was equivalent to that of wild type (WT) virus in RIPK3 knockout mice [119]. The growth defect of the M45 mutant could also be rescued by infecting mice lacking DAI/ZBP1 [41] or expressing a Z-RNA binding-deficient DAI/ZBP1 protein [63]. This elegant series of observations established that the DAI/ZBP1 PRR senses Z-RNA produced during MCMV infection resulting in activation of RIPK3-dependent necroptosis (Figure 2), and that successful replication of MCMV depends upon suppression of this process by M45. Similarly, M45 can block TLR3 and TLR4-induced necroptosis by inhibiting the RHIM-dependent activation of RIP3 by the adaptor protein TRIF [42] (Figure 2).

Besides inhibiting necroptosis, M45 also modulates TNFR-dependent activation of transcription factor NF-κB and p38 mitogen-activated protein kinase by interacting with RIPK1 [117,120]. Moreover, M45 also interacts with the NF-κB essential modulator (NEMO) and redirects it to autophagosomes for degradation [121]. By blocking all canonical NF-κB activating pathways, M45 inhibits NF-κB-dependent expression of proinflammatory cytokines and survival factors. Thus, M45 has multiple functions that affect cell death, survival, and inflammation.

How the processes of apoptosis and necroptosis interact during viral infection was recently investigated using an MCMV mutant lacking functional M36 and M45. Macrophages infected with the double mutant activated caspase-8 to drive apoptosis early, with cells then progressing to secondary RIPK3-dependant necroptosis [122]. This form of cell death was highly inflammatory, resulting in an improved T cell response after in vivo infection. This data suggests that the simultaneous suppression of apoptosis and necroptosis not only prevents the premature death of infected cells, but also serves as a means of restricting the inflammatory response.

HCMV infection is also known to block the induction of necroptosis [123]. In contrast to MCMV, inhibition of necroptosis by HCMV occurs after the activation of RIPK3 and phosphorylation of MLKL. The viral protein responsible for conferring protection is regulated by IE1, but its identity has not been determined [123]. Interestingly, HCMV UL45 differs from its MCMV homologue, M45, in that it does not contain a RHIM. In contrast, the homologous proteins in herpes simplex virus (HSV) type 1 and 2 carry a RHIM [124]. The HSV1 ICP6 protein inhibits necroptosis in human cells [125], but surprisingly activates necroptosis in murine cells [126,127]. These results have established that necroptosis is an evolutionarily conserved response to herpesvirus infection. RHIM-mediated inhibition of necroptosis is a conserved strategy of both MCMV and HSV (at least in cells of their natural host), but MCMV and HCMV have developed distinct methods for antagonizing this process.

### 4.5. Interference with Pyroptosis?

CMV infection is known to activate the inflammasome [65]. If CMV can suppress this pathway is unclear, but some evidence from in vitro experiments suggests this may be the case. Expression of HCMV UL83 was sufficient to cause a reduction in the expression of AIM2 and inhibit the processing of IL-1β [128]. A reduction in the expression level of pro-IL-1β following MCMV infection has also been noted [129]. While tantalising, further work is required to determine if CMV has any significant capacity to interfere with pyroptosis.

## 5. Concluding Remarks

Mammalian cells have evolved an array of mechanisms to detect the presence of intracellular pathogens, with many of these pathways culminating in the activation of the cell death response. The study of CMV mutants has clearly established that successful viral replication requires the capacity to curb multiple host cell death pathways. While many of the viral inhibitors are conserved between HCMV and MCMV, some differences in how the respective viruses prevent cell death have been noted. For example, both viruses prevent necroptosis but they do so by targeting different points in the pathway. Indeed, the divergent anti-death strategies used by the various CMVs are proposed as one of the reasons why these viruses are so highly species specific [130].

Inhibition of cell death not only affords CMV an opportunity to complete the replication cycle, but also restricts the inflammatory response required for the generation of the adaptive immune response. Preventing cell death therefore serves several functions that contribute to viral pathogenesis. CMV-derived inhibitors of all the major cell death pathways have been described, with the exception of pyroptosis. If CMV inhibits the process of pyroptosis to any significant extent, and how this contributes to viral infection, is one of the significant questions that remains to be addressed. A second unresolved issue is if CMV latency or reactivation requires the inhibition of cell death. In support of this possibility, CD34^+^ progenitors harbouring a latent HCMV infection exhibit an increased resistance to apoptosis [131]. The elucidation of the mechanisms used by CMV to prevent cell death has not only enhanced our understanding of viral pathogenesis but provided insights into how host cell death responses are regulated. An exciting possibility is that this knowledge could be used to develop improved treatments for HCMV infections, a virus that causes significant morbidity and mortality in immunosuppressed patients.
